# Assessing the Impact of Transgenerational Epigenetic Variation on Complex Traits

**DOI:** 10.1371/journal.pgen.1000530

**Published:** 2009-06-26

**Authors:** Frank Johannes, Emmanuelle Porcher, Felipe K. Teixeira, Vera Saliba-Colombani, Matthieu Simon, Nicolas Agier, Agnès Bulski, Juliette Albuisson, Fabiana Heredia, Pascal Audigier, David Bouchez, Christine Dillmann, Philippe Guerche, Frédéric Hospital, Vincent Colot

**Affiliations:** 1 Unité de Recherche en Génomique Végétale, Centre National de la Recherche Scientifique (CNRS) UMR 8114, Institut National de la Recherche Agronomique (INRA) UMR 1165, Université d'Evry Val d'Essonne, Evry, France; 2 Institut Jean-Pierre Bourgin, Station de Génétique et d'Amélioration des Plantes UR 254, INRA, Versailles, France; 3 Laboratoire de Physique Théorique et Modèles Statistiques, CNRS UMR 8626, Université Paris-Sud, Orsay, France; 4 Groningen Bioinformatics Centre, University of Groningen, Haren, The Netherlands; 5 Ferme du Moulon, Université Paris-Sud, INRA, UMR 0320/UMR 8120, Génétique Végétale, Gif-sur-Yvette, France; 6 CNRS UMR 8186, Département de Biologie, Ecole Normale Supérieure, Paris, France; 7 INRA, UMR 1313 Génétique Animale et Biologie Intégrative, Jouy-en-Josas, France; Queensland Institute of Medical Research, Australia

## Abstract

Loss or gain of DNA methylation can affect gene expression and is sometimes transmitted across generations. Such epigenetic alterations are thus a possible source of heritable phenotypic variation in the absence of DNA sequence change. However, attempts to assess the prevalence of stable epigenetic variation in natural and experimental populations and to quantify its impact on complex traits have been hampered by the confounding effects of DNA sequence polymorphisms. To overcome this problem as much as possible, two parents with little DNA sequence differences, but contrasting DNA methylation profiles, were used to derive a panel of epigenetic Recombinant Inbred Lines (epiRILs) in the reference plant *Arabidopsis thaliana*. The epiRILs showed variation and high heritability for flowering time and plant height (∼30%), as well as stable inheritance of multiple parental DNA methylation variants (epialleles) over at least eight generations. These findings provide a first rationale to identify epiallelic variants that contribute to heritable variation in complex traits using linkage or association studies. More generally, the demonstration that numerous epialleles across the genome can be stable over many generations in the absence of selection or extensive DNA sequence variation highlights the need to integrate epigenetic information into population genetics studies.

## Author Summary

DNA methylation is defined as an epigenetic modification because it can be inherited across cell division. Since variations in DNA methylation can affect gene expression and be inherited across generations, they can provide a source of heritable phenotypic variation that is not caused by changes in the DNA sequence. However, the extent to which this type of phenotypic variation occurs in natural or experimental populations is unknown, partly because of the difficulty in teasing apart the effect of DNA methylation variants (epialleles) from that of the DNA sequence variants also present in these populations. To overcome this problem, we have derived a population of epigenetic recombinant inbred lines in the plant *Arabidopsis thaliana*, using parents with few DNA sequence differences but contrasting DNA methylation profiles. This population showed variation and a high degree of heritability for two complex traits, flowering time and plant height. Multiple parental DNA methylation differences were also found to be stably inherited over eight generations in this population. These findings reveal the potential impact of heritable DNA methylation variation on complex traits and demonstrate the importance of integrating epigenetic information in population genetics studies.

## Introduction

Continuous trait variation in natural and experimental populations is usually attributed to the actions and interactions of numerous DNA sequence polymorphisms and environmental factors [1]. These so-called complex traits encompass many of the prevalent diseases in humans (e.g. diabetes, cancer) as well as many agriculturally and evolutionarily important traits (e.g. yield, drought resistance, or flowering time in plants). The heritable basis of complex traits is classically thought to rest solely on the transmission from parents to offspring of multiple DNA sequence variants that are stable and causative [1]. However, accumulating evidence suggests that this view may be too restrictive, insofar as chromatin variation (such as differential DNA methylation) can also be propagated across generations with phenotypic consequences, independent of DNA sequence changes [2]–[7]. Indeed, examples of spontaneous, single-locus DNA methylation variants (epialleles) have been reported to influence a range of characters, such as flower shape or fruit pigmentation in plants [8],[9] and tail shape or coat color in the mouse [10],[11]. By extension, these observations raise the possibility that the genome-wide segregation of multiple epialleles could provide a so far unexplored basis of variation for many commonly studied complex traits [12].

In the flowering plant *Arabidopsis thaliana*, recent large-scale DNA methylation profiling has revealed a substantial degree of differences between natural accessions [13],[14]. As these accessions also differ in their DNA sequences, experimental populations derived from them, such as backcrosses, F2-intercrosses or Recombinant Inbred Lines (RILs) could potentially segregate two independent sources of heritable phenotypic variation, which are difficult to disentangle from each other [12]. As a consequence of this confounding issue, there has been little effort to date to quantify the impact of epigenetic factors on complex traits and to assess their role in the creation and maintenance of phenotypic diversity in experimental or natural settings [2],[6].

To overcome this problem as much as possible, we established a population of epigenetic Recombinant Inbred Lines (epiRILs) in Arabidopsis. This population was derived from two near-isogenic parental lines, one wild type (wt) and the other mutant for the *DDM1* gene. *DDM1* encodes an ATPase chromatin remodeler that is primarily involved in the maintenance of DNA methylation and silencing of repeat elements [15]–[18]. Thus, *ddm1* mutant plants exhibit a ∼70% reduction of DNA methylation overall, as well as a widespread over-accumulation of transcripts corresponding to transposable elements (TEs) [15],[16],[18]. Despite this, few TEs appear to show increased transposition in *ddm1* [19],[20], perhaps as a result of many TEs still being targeted by the RNAi-dependent DNA methylation machinery in this mutant background [21]. Consistent with these molecular properties, *ddm1* plants exhibit only mild phenotypic alterations, except after repeated selfing, in which case the severity and the number of aberrant phenotypes tend to increase [22]. Genetic analysis has shown that many of these phenotypes segregate independently of the *ddm1* mutation and are conditioned by recessive or dominant alleles of single loci. Furthermore, molecular characterization of five of these alleles indicated that they arose through TE-mediated gene disruption in one case [19] and through late onset epigenetic alteration of gene expression in the other cases, often in the context of genes that are tightly associated with TE sequences [19], [22]–[27]. Based on these observations, and given a constant environment, variation in complex traits between epiRILs is expected to result from the stable inheritance of multiple epigenetic differences (epialleles) induced by *ddm1* and/or from a small number of DNA sequence differences that might also be present between epiRILs, notably as a result of *ddm1*-induced mobilization of some TEs.

Here, we describe the phenotypic analysis of the epiRIL population, which revealed a high degree of heritability for flowering time and plant height. We also show that the epiRILs differ by numerous parental epialleles across the genome, which demonstrates that DNA methylation differences can be stably inherited over at least eight generations in the absence of extensive DNA sequence polymorphisms and with no selection. These findings provide a first indication of the potential impact of epigenetic variation on complex traits.

## Results

### Construction of the Col-wt EpiRILs

**Figure 1:**
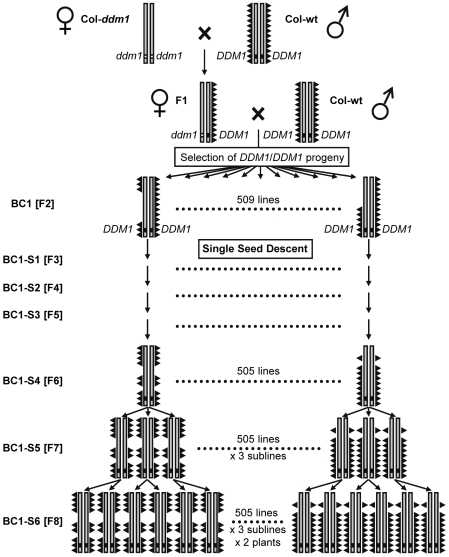
Construction of the Col-wt epiRILs. Grey bars represent the *A. thaliana* genome, and triangles represent DNA methylation. Except at the *DDM1* locus (black and white squares) located on chromosome 5, the two parents (Col-wt and Col-*ddm1*) are near isogenic; they differ however in their levels of DNA methylation. An F1 individual was backcrossed to the Col-wt parental line, and 509 *DDM/DDM1* BC1 individuals were selfed. After three more selfing (BC1-S4), three independent sublines were established and selfed once to obtain the Col-wt epiRIL population (See Materials and Methods).

The epiRIL population was initiated using two closely related parents of the same accession (Columbia, Col), one homozygous for the wild type *DDM1* allele (Col-wt), and the other for the *ddm1-2* mutant allele (Col-*ddm1*, 4^th^ generation). Therefore, these two parents should differ extensively in their DNA methylation profiles [18], but only marginally in their DNA sequence, namely at the *DDM1* locus itself and at a few other sites, such as those affected by *ddm1*-induced mobilization of transposable elements (see Materials and Methods and below). A single F1 plant was backcrossed as female parent to the Col-wt parental line. From the backcross progeny, we selected over 500 individuals of *DDM1*/*DDM1* genotype, from which a final population of 505 Col-wt epigenetic Recombinant Inbred Lines (Col-wt epiRILs) were derived through six rounds of propagation by single seed descent and no selection bias (Figure 1; Materials and Methods). The Col-wt epiRILs should therefore have highly similar genomes, but markedly distinct epigenomes, if the many DNA methylation variants induced by *ddm1* are stably inherited.

### The Col-wt EpiRILs Show Heritable Variation for Two Quantitative Traits

**Figure 2:**
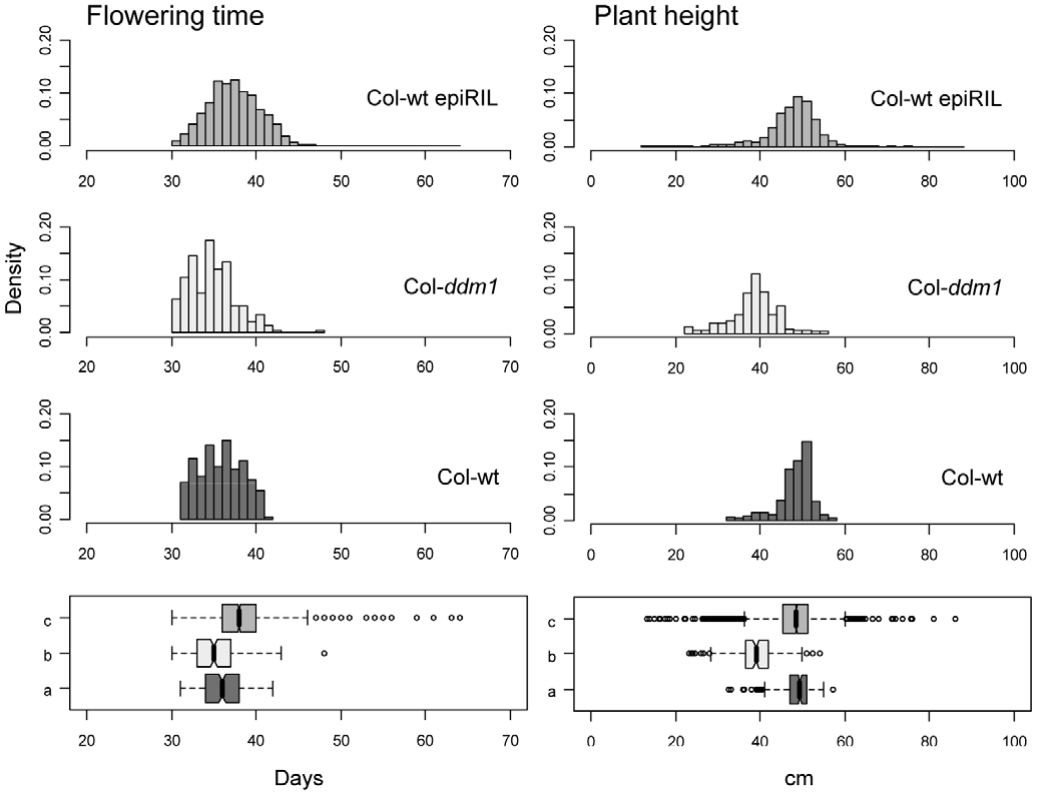
Phenotypic distributions. Top panels: density histograms of raw phenotypic values for flowering time and plant height for the Col-*ddm1* parental line, the Col-wt epiRILs, and the Col-wt parental line. The units on the x-axis are given in *days* and *cm* for these traits, respectively; the y-axis shows the density. Bottom panels: box-whisker plots for the three populations (a: Col-wt parental line; b: Col-*ddm1* parental line; c: Col-wt epiRILs) with sample median; the whiskers mark off ±3 standard deviations from the mean; outlier data points are represented by open circles. A total of 16 individual Col-wt epiRIL plants were outliers (>3SD) for flowering time and 52 for plant height. These outliers mainly belong to a few Col-wt epiRILs lines (3 and 8 for flowering time and plant height, respectively) and were removed for subsequent heritability analysis.

**Figure 3:**
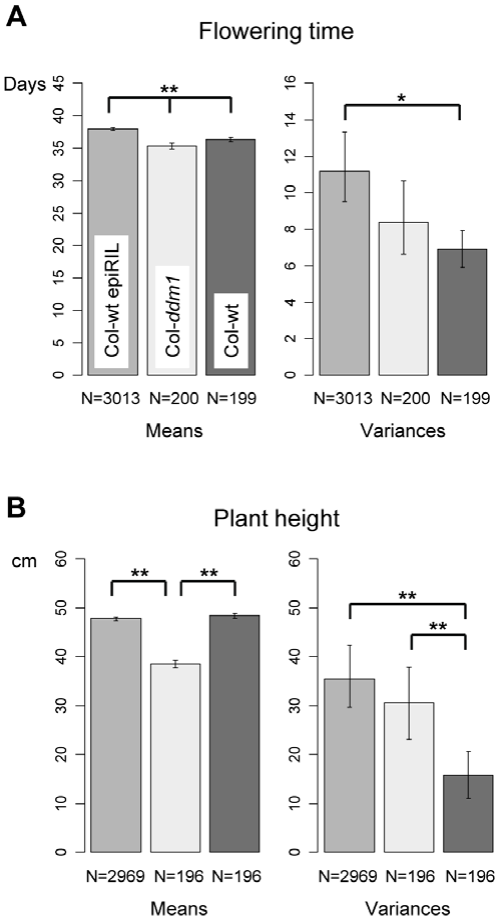
Comparison of phenotypic means and variances. (A) Flowering time. (B) Plant height. The different populations are color-coded as indicated in the top left panel. * *p*-value (*p_B_*)<0.05; ** *p*-value (*p_B_*)<0.01; The effective sample sizes are indicated below each bar plot.

Phenotypic analysis of the Col-wt epiRILs was performed for two quantitative traits, flowering time and plant height at maturity (Table S1). As illustrated in Figure 2 and Figure 3, larger phenotypic variation was observed among the Col-wt epiRILs, than among the Col-wt or Col-*ddm1* parental lines (see also Tables S2, S3, S4). Increased phenotypic variation of this kind is indicative of a component of segregational variance that typically arises in the construction of Recombinant Inbred Lines obtained from parents that differ by numerous DNA sequence polymorphisms [1], except that in the present design the two parents are expected to be nearly isogenic.

**Figure 4:**
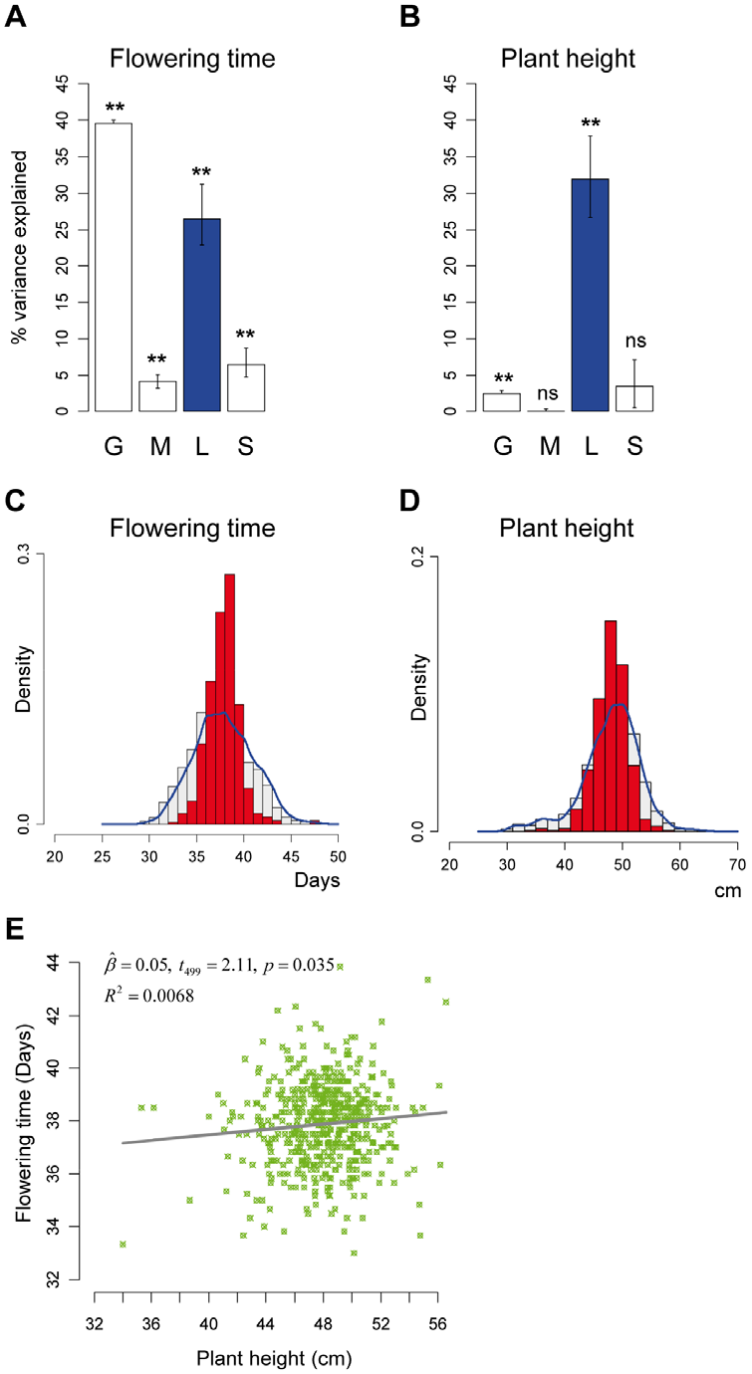
Estimates of heritable phenotypic variance. (A,B) Percent of phenotypic variance explained by each of the tested variables and their 95% confidence intervals; G = Greenhouse effect; M = Micro-environment effect; L = Line-effect; S = Subline-effect. The effective samples sizes were 2856 and 2813 for flowering time and plant height, respectively. Outliers (>3SD) were removed from the analyses. (C,D) For the two traits, density histograms (red) of Col-wt epiRILs line means (‘genetic’ values) are superimposed over a density histogram of the total phenotypic variation (grey histogram with blue density line). By visual inspection, the distribution of the line means is continuous, suggestive of ‘polygenic’ variation for these traits. (E) Bivariate plot and least-squares fit (black line) of Col-wt epiRILs line means between plant height (*x*-axis) and flowering time (*y*-axis) reveals a negligible ‘genetic’ correlation, suggesting that these two traits have a largely distinct heritable basis; * *p*-value<0.0001; ns = not significant at *α* = 0.05 (Table S5).

To decompose the sources of phenotypic variation observed among the Col-wt epiRILs, a series of linear mixed models were fitted (Materials and Methods and Table S5). As in classical quantitative genetics analysis, the estimated between-line variance (line-effect) gives a direct estimate of broad-sense heritability, *i.e.* the fraction of phenotypic variance that is not due to environmental effects (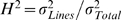; ref [1]). Large and significant heritability values were obtained for flowering time (0.26, *p*<0.0001; Figure 4A; Table S5) and plant height (0.32, *p*<0.0001; Figure 4B; Table S5). The fact that the means (‘genetic’ values) of the Col-wt epiRILs for the two traits appear to follow a continuous distribution (Figure 4C and 4D) suggests that both traits are subject to a “polygenic” rather than a single locus inheritance model. Moreover, the line means of flowering time and plant height are only weakly correlated with each other (Figure 4E). This observation points towards a distinct heritable basis for these two traits, and indicates that the two heritability estimates obtained here are not redundant.

The excess of variance and the high heritability values observed in the population of Col-wt epiRILs could be caused by (i) segregation of multiple parental epialleles, (ii) segregation of parental differences in DNA sequence created by *ddm1*-induced mobilization of transposable elements, and (iii) mutation or epimutation accumulation in the Col-wt epiRILs as a result of selfing over multiple generations. We explored the latter possibility by first comparing the heritability estimates obtained for the Col-wt epiRIL population with those from a panel of 24 Col-wt control lines (N = 144) that were derived from the Col-wt parent and propagated along with the Col-wt epiRILs through six rounds of single-seed descent (see Materials and Methods and Text S1). The heritability estimates obtained in these control lines were negligible for flowering time (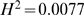) and plant height (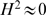) and significantly lower compared to those of the Col-wt epiRILs (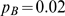; 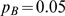, respectively; Text S1).

Furthermore, sublines that were derived at the F7 generation (Figure 1) of the Col-wt epiRIL design made only a small contribution to the total phenotypic variance (Figure 4A and 4B), suggesting that epimutation accumulation or increased mutation rate (notably through continuing transposon mobilization, [28]; see below) contribute minimally. Although the subline effect for flowering time does explain about 6% of the variance, this estimate is not specific to a source of new (epi)mutational variance but rather reflects a compound estimate that also includes gene x environment interactions as well as maternal effects. Hence, based on these subline estimates, novel DNA sequence or methylation variants that could have arisen during the selfing of the Col-wt epiRILs appear to have little phenotypic consequences. This conclusion is also supported by the very small number of lines that were lost during the construction of the Col-wt epiRILs (4 out of 509; Materials and Methods), and by the limited number of outliers (±3SD) for the two complex traits considered (Figure 2, bottom panels). This contrasts with the progressive phenotypic degeneracy that has been observed upon repeated selfing of *ddm1* mutant plants [22].

Evidence for a stable heritable basis of both flowering time and plant height also comes from the observation that the phenotypic means in the Col-wt epiRILs are in each case closer to the Col-wt than to the Col-*ddm1* parental mean (Figure 2). This is of course entirely consistent with the backcross scheme used to derive the Col-wt epiRILs (Figure 1). Taken together, these findings provide evidence that heritable variation for flowering time and plant height in the Col-wt epiRIL population is due to the stable inheritance and segregation of parental epialleles and/or DNA insertion variants, rather than the accumulation of new mutations or epimutations.

### Numerous Parental DNA Methylation Variants Are Inherited in the Col-wt EpiRILs

**Figure 5:**
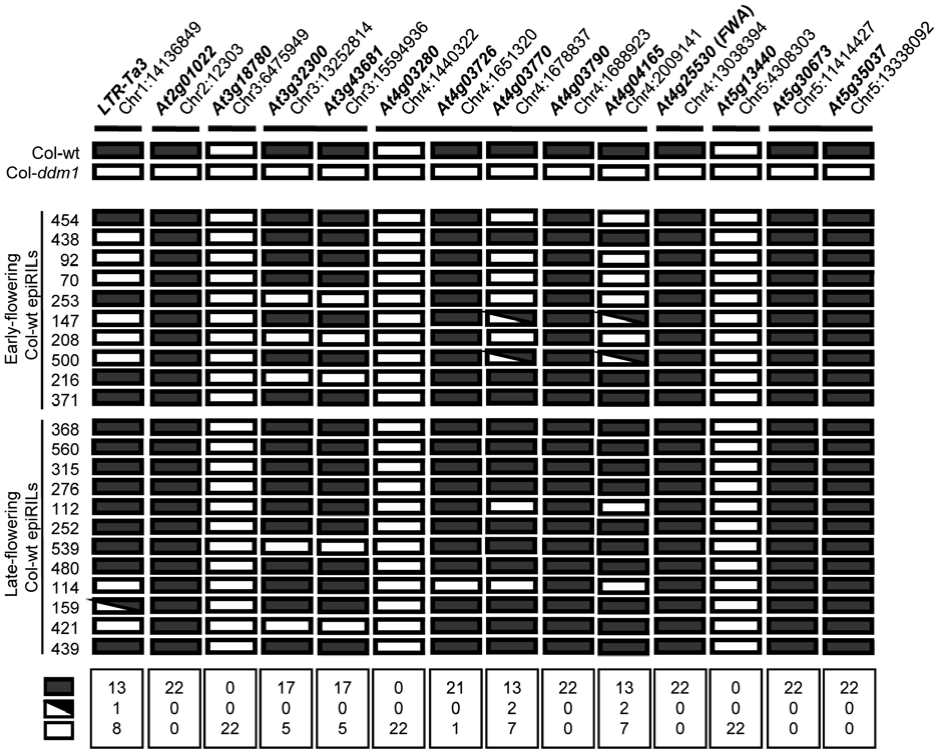
Segregation analysis of DNA methylation in a sample of early and late flowering Col-wt epiRILs. Name and position of probed sequences are indicated at the top. Horizontal bars underneath indicate closely linked sequences. Black and white rectangles represent high (wt) methylation, and absence of methylation or *ddm1*-induced hypomethylation, respectively. Sectored rectangles represent intermediate methylation levels between Col-wt and Col-*ddm1* (for examples of actual methylation measurements, see Figure S1). Segregation of methylation states among the 22 Col-wt epiRILs (F9) is summarized for each sequence at the bottom of the graph.

The inheritance of parental epialleles was tested by analyzing the methylation state of several loci in a number of Col-wt epiRILs. Genomic DNA was digested with the enzyme McrBC, which only cuts methylated DNA, and specific sequences were amplified by real-time PCR (Text S1). Eleven sequences were chosen that are methylated in the Col-wt and hypomethylated in the Col-*ddm1* parental lines (Figure 5, Figure S1), including the *FWA* gene, for which hypomethylation and ectopic expression have been associated with a large delay in flowering [24],[29]. Additionally, three control sequences were chosen that are not methylated in either of the two parents (Figure 5, Figure S1). Twenty-two Col-wt epiRILs were sampled at the F9 (BC1-S7) generation from both ends (but excluding outliers, see Figure 2) of the flowering time distribution (Figure 5). Results were consistent with the three non-methylated parental sequences being stably inherited in their non-methylated state in the Col-wt epiRILs, and with five of the eleven differentially methylated parental sequences segregating in a Mendelian or near-Mendelian manner (72.8% [∼16/22] met./met., 0.4% [∼0/22] met./hypomet., 26.8% [∼6/22] hypomet./hypomet. at BC1-S7; Figure 5 and Text S1). In contrast, the other six sequences that were differentially methylated in the parental lines, including *FWA*, did not segregate in the Col-wt epiRILs. Rather, these sequences were found in the fully methylated state in all 22 lines, except for the At4g0376 sequence, which was unmethylated in one line (Figure 5). These results confirm and extend those of our previous analysis which indicated that while some hypomethylated epialleles induced by *ddm1* are stably inherited over at least eight generations, others efficiently regain wt DNA methylation within two to five generations following restoration of DDM1 function, as a result of being targeted by the RNAi-dependent DNA methylation machinery [21].

Taken together, these findings provide evidence that the stable inheritance and segregation of parental epialleles is likely involved in the heritable variation for flowering time and plant height in the Col-wt epiRIL population. Furthermore, the efficient DNA remethylation of a subset of *ddm1*-induced epialleles could partly explain the closer proximity of the Col-wt epiRIL phenotypic means to those of Col-wt parental line (Figure 3).

### The *FWA* Locus Contributes Marginally to the Continuous Variation for Flowering Time Observed in the Col-wt EpiRIL Population

**Figure 6:**
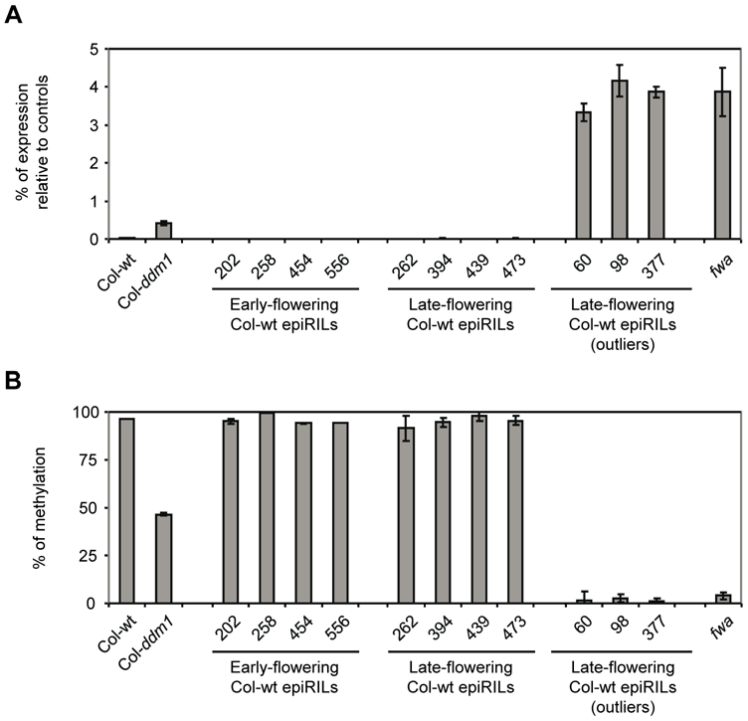
DNA methylation and expression analysis of the *FWA* locus. (A) McrBC-QPCR analysis of DNA methylation. (B) RT–QPCR analysis of transcript levels. Results are expressed as % of expression relative to the average of three control genes (see Materials and Methods).

Previous studies have shown that *ddm1*-induced hypomethylation and ectopic expression of *FWA* can be stably inherited over many generations independently of the *ddm1* mutation and cause severe delay in flowering time [23],[24]. However, our observation that *FWA* had wt DNA methylation levels in all 22 Col-wt epiRILs analyzed, which included 12 late flowering lines (Figure 5), suggested instead efficient RNAi-mediated DNA remethylation of this locus, and therefore at best a marginal contribution of *FWA* to the continuous variation for flowering time in the Col-wt epiRIL population. To explore this further, *FWA* methylation and expression were measured for an additional set of four early and four late flowering lines that fall within three standard deviations from the mean (38±10 days, Figure 2), as well as for the three late flowering outlier lines (>48 days, Figure 2) that are present in Col-wt epiRIL population. While *FWA* methylation and expression were indistinguishable from wt in all of the non-outlier lines, hypomethylation was observed in the three late flowering outlier lines and was associated with high-level expression in seedlings, where the gene is normally not expressed (Figure 6). Moreover, *FWA* hypomethylation and transcript accumulation in these outlier lines were much more pronounced than in the Col-*ddm1* parental line and were similar to those of a previously described, *ddm1*-induced late flowering line (Figure 6; [23],[24]). Thus, while the *FWA* allele of the Col-*ddm1* parent was efficiently remethylated and resilenced upon restoration of DDM1 function, further hypomethylation and reactivation occurred instead in rare cases, leading to overtly late flowering Col-wt epiRILs. These results confirm that epiallelic variation at *FWA* has a major effect on flowering time, but indicate also that it is rare in the Col-wt epiRIL population, concerning phenotypic outliers that were removed from the quantitative genetics analysis. We conclude therefore that epiallelic variation at *FWA* contributes little to the continuous variation in flowering time observed in the Col-wt epiRIL population.

### Mobilization of Transposable Elements Occurs in the *ddm1* Parental Line and the Col-wt EpiRILs

**Figure 7:**
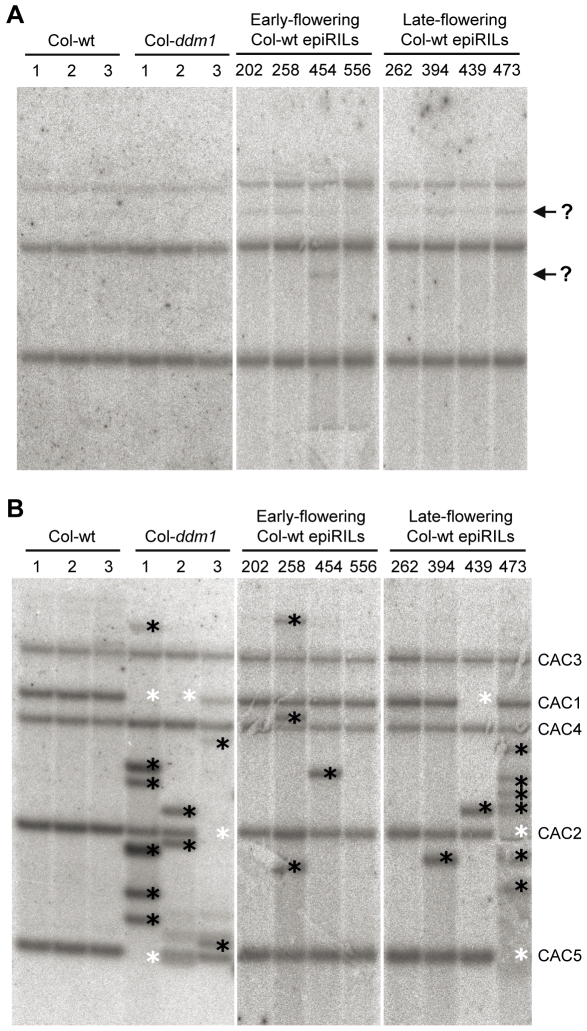
Southern blot analysis of TE mobilization. (A,B) Southern blot analysis of two TE families (*MULE* and *CACTA*, respectively) in three individuals of each parental line (Col-wt and Col-*ddm1*), as well as in four early- and four late-flowering Col-wt epiRILs (F9). Genomic DNA was digested using *Hin*dIII and hybridized after gel electrophoresis and transfer to a nylon membrane using previously described probes [19],[20]. Question marks indicate possible new insertion sites for *MULE*. For CACTA, white and black stars designate excision events and new insertions, respectively. The five *CACTA* copies present in wt Columbia [42] are indicated on the right.

Apart from epialleles, DNA sequence variants caused by *ddm1*-induced transposon mobilization could also segregate among the epiRILs. To test this possibility, we carried out Southern blot analysis of the insertion profile of *CACTA* and *MULE* transposons, which are the two TE families for which *ddm1*-induced mobility has been documented [19],[20]. Little transposition was detected for any of the three *MULE* copies in either the three *Col-ddm1* individuals or the eight Col-wt epiRILs that were analyzed (Figure 7A). In contrast, several transposition events could be detected for *CACTA* in the individuals of the Col-*ddm1* parental line as well in the Col-wt epiRILs. More specifically, excision events were observed for three of the five CACTA copies that are present in wt Columbia, as indicated by the disappearance of the corresponding hybridizing fragments (Figure 7B, white asterisks). In addition, new insertions were detected, in the form of new hybridizing fragments (Figure 7B, black asterisks). The observation of continuing *CACTA* mobilization in the Col-wt epiRILs is consistent with previous results indicating that *CACTA* copies remain transpositionnally active following restoration of wild type DDM1 function through backcrosses [28]. This highly mobile transposon family may therefore contribute to the heritable variation observed among the Col-wt epiRILs, although no obvious association between specific *CACTA* insertion differences and flowering time variation could be detected based on our limited sampling (Figure 7B).

## Discussion

Using a population of “epigenetic” Recombinant Inbred Lines (epiRILs) in the flowering plant *Arabidopsis thaliana*, we have demonstrated that multiple DNA methylation changes induced across the genome can be stably inherited over at least eight generations in the absence of selection, and that these changes were associated with substantial heritable variation in two complex traits. Furthermore, we show that epiallelic variation at the *FWA* locus has a major effect on flowering time but is rare in our epiRIL population, indicating that other loci are involved in the continuous variation for that trait in this population.

In practical terms, our findings pave the way for the identification of causative epigenetic quantitative trait loci (*ph*QTL*^epi^*; [12]) in the Col-wt epiRIL population using whole genome DNA methylation profiling and classical linkage mapping methods, without the confounding effect of widespread DNA sequence polymorphisms [12]. By combining bisulphite methodology to interrogate the methylation status of individual cytosines with next generation sequencing [30],[31], it may now be possible to identify simultaneously the epigenetic variants segregating in the Col-wt epiRIL population and the inevitable rare DNA sequence variants also present in this population, notably as a result of *ddm1*induced transposable element mobilization (Figure 7). Alternatively, epigenotyping and genotyping could be carried out independently, using immunoprecipitation of methylated DNA (MeDIP) followed by hybridization to whole genome tiling arrays and next generation sequencing, respectively.

The heritability values (around 30%) obtained in our study are similar to those considered in classical breeding programs for the improvement of agronomic traits. If QTL mapping of the Col-wt epiRILs were to confirm that heritability is largely due to variations in DNA methylation states, the view that DNA sequence variation is the sole basis of the heritability of complex traits may need to be revised substantially. In addition, QTL mapping will provide valuable insights into how epigenetic variation can modulate the rate of DNA sequence change in a population, notably through TE mobilization.

In the context of evolutionary biology, the existence of an additional mechanism for the creation of heritable variation in complex traits could explain the faster than expected adaptation to environmental change that is often observed in natural populations [32]. There is indeed mounting evidence that epigenetic alterations (epimutations) can arise at high frequency, in response to environmental challenges or ‘genomic shocks’ [5],[33],[34]. Furthermore, our findings provide clear evidence that many epigenetic variants can be stably inherited over numerous generations in the absence of selection ([21]; this study). Such stability could thus provide populations with sufficient time to explore the adaptive landscape [35], and for neutral mutations to accumulate over the new epialleles, in a process that could ultimately lead to genetic assimilation [36].

On the other hand, the observation that about one half of DNA hypomethylation variants induced by *ddm1* systematically regain wt DNA methylation over two to five generations ([21]; Figure 5) illustrates the potentially transient nature of many epialleles. However, analysis of *FWA* indicates that even in the case of these so-called remethylatable alleles, stable transmission of hypomethylated (and reactivated) states can occur at low frequency (Figure 6). Indeed, our findings are consistent with previous observations of sporadic occurrence of stable, phenotypic *FWA* hypomethylated epialleles (*fwa*) in *ddm1* mutant lines [23]. Furthermore, comparison of *FWA* methylation and expression levels between the *Col-ddm1* parental line and *fwa* as well as Col-wt epiRIL late flowering outliers suggests that stable transmission of hypomethylated/reactivated *FWA* can only occur when specific thresholds of hypomethylation/reactivation are reached (Figure 6A). Finally, although no naturally hypomethylated *FWA* epiallele has been recovered in a survey of 96 Arabidopsis accessions [13], it is tempting to speculate, on the basis of our observations at this locus, that the varying stability of epialleles could underlie the variable penetrance of disease-causing alleles that segregate in pedigrees, as well as the variable onset of many heritable diseases in response to developmental or environmental cues [37].

In summary, our study provides important new evidence that epigenetic variation can contribute significantly to complex traits, and lays the foundation for identifying causative loci. The conditions that promote the occurrence of epialleles and their transgenerational stability in natural settings will need to be further elucidated in order for epigenetics to be fruitfully incorporated into the quantitative genetic analysis of experimental and natural populations [12].

## Materials and Methods

### Construction of the Col-wt EpiRILs and Col-wt Control Lines

The recessive *ddm1-2* mutation was isolated in a screen for marked decrease in DNA methylation of centromeric repeats in EMS-mutagenized seeds of the Columbia (Col) accession [16]. The Col-wt and Col-*ddm1* parental lines were both derived from a *ddm1/DDM1* plant stock that had been maintained in the heterozygous state by repeated backcrossing to a wild type Columbia line over six generations to remove EMS-induced mutations unlinked to *ddm1* (a kind gift from Eric Richards, Washington University, Saint Louis, MO, USA). Homozygous *DDM1/DDM1* and *ddm1/ddm1* progeny were subsequently selfed for four generations. In *ddm1*/*ddm1* plants, this generated genome-wide DNA hypomethylation as well as mobilization of some transposable elements ([16], [18]–[20]; Figure 7). A single plant of each genotype (Col-wt and Col-*ddm1*) was then used for the initial Col-wt epiRIL cross (Figure 1). Unlike in classical RIL construction, the two parents were thus near isogenic, being derived from siblings that underwent four generations of selfing, but differed extensively in their levels and patterns of DNA methylation. The Col-*ddm1* parent that was used to initiate the Col-wt epiRIL cross looked normal and did not display any of the developmental epimutant phenotypes that have been reported in advanced *ddm1* lines, such as *superman* [27], *fwa* [23],[24], *ball* [22],[25], or *bonsai* [26]. A single F1 individual was backcrossed to the Col-wt parental line (Figure 1). The BC1 progeny was screened by PCR-based genotyping (Text S1): of the 1140 BC1 individuals genotyped, 577 were *ddm1/DDM1*, 521 were *DDM1/DDM1*, and 42 were *ddm1/ddm1*. This last genotype was indicative of low-level contamination of the backcross progeny with seeds produced by self-pollination of the female F1 parent. Indeed, subtracting 42 and 84 potential self-pollination contaminants from the *DDM1/DDM1* and *ddm1/DDM1*genotypic classes, respectively, gives a corrected total of 479 *DDM1/DDM1* and 493 *ddm1/DDM1* individuals, close to the 1∶1 ratio expected for the backcross. Only the *DDM1/DDM1* individuals were considered for the construction of the Col-wt epiRILs (Figure 1), and our calculations show that this amount of contamination (42 out of 521 or 8% of *DDM1/DDM1* BC1 individuals) has a negligible effect on the expected epigenotype frequencies in subsequent generations (Text S1). In total, 509 out of the 521 *DDM1/DDM1* BC1 individuals were selfed and one seedling per line was randomly retained from four seeds sown. This process was repeated at each of the following generations (single seed descent (SSD) approach) and ensured that seedlings could be recovered in most instances with no selection bias. Under the assumption of epiallelic stability, each of the *DDM1/DDM1* BC1 founders should have inherited from the female F1 parent, on average, 50% of the transmissible DNA methylation alterations that were present in the *ddm1/ddm1* grandparent (Figure 1). This should lead, after repeated selfing, to the inheritance of an average of 25% of these alterations in each Col-wt epiRIL, except of course for the 8% of Col-wt epiRILs expected to derive from self-pollination of the female F1 parent, which should have each inherited instead 50% of these alterations on average. Four Col-wt epiRILs were lost during propagation and each of the remaining 505 Col-wt epiRILs was subdivided into three sublines at the F6 generation (Figure 1) to obtain 3×505 BC1-S5 (F7) plants. These were again selfed, and two BC1-S6 (F8) individuals per subline were retained for the phenotypic and quantitative genetics analyses. Since the *ddm1* mutation is recessive, it follows that the sublines obtained at BC1-S6 had been free of the conditioning *ddm1* mutant allele effect for a total of 8 generations.

We also established 24 Col-wt control lines, starting from 24 full-sib individuals of the Col-wt parental line (hence of the same genetic background as the Col-wt epiRILs). These control lines were propagated by repeated SSD, and subdivided into three sublines before phenotypic analyses, using the same method as described above with the Col-wt epiRILs (Figure 1).

### Experimental Conditions and Phenotype Measurements

The Col-wt epiRILs (N = 3030), the Col-wt control lines (N = 144), the Col-wt (N = 200) and Col-*ddm1* (N = 200) parental populations were grown simultaneously in two replicate climate-controlled greenhouses under long day conditions (day: 16 h - 20°C/22°C, night: 8 h - 16°C/18°C) with complement of artificial light (105 µE/m^2^/s) when necessary. For the Col-wt epiRILs, one of the two *BC1-S6* plants for each subline was grown in each greenhouse (i.e. 3×505 Col-wt epiRIL plants in each greenhouse). Within each greenhouse, the Col-wt epiRIL plants were randomized over 28 tables (3×1 m^2^). In addition, two or three plants from each parental line were systematically placed on each table. Finally, the positions of Col-wt epiRILs and parental lines were randomized within tables. Plants were grown in individual pots (7×7×7 cm^3^) filled with a 90∶10 mix of peat and volcanic sand, and topped with a thin layer of granulated cork. About 15 seeds were sown per pot and seedlings were thinned out to retain a single plant that appeared representative of the whole family. Plants were supplemented twice with a nutritive solution during the reproductive phase. Of the planned design, >99% of plants were available for trait measurements. Flowering time (i.e. number of days between sowing and opening of the first flower) was recorded during plant growth. When plants ceased flowering, they were harvested and stored in herbaria. Plant height was then measured on the dried plants.

### Statistical Analysis

Phenotypic means and variances were calculated for the Col-wt and Col-*ddm1* parental lines, the Col-wt epiRILs and the Col-wt control lines. The corresponding 95% confidence intervals were obtained empirically from 3000 non-parametric bootstrap draws. For the Col-wt epiRIL and Col-wt control populations, in which individual plants were phenotypically more similar than plants taken at random, a stratified bootstrap approach was implemented where each line was taken as an independent stratum. In this way, the boostrap estimates are consistent with the stochastic structure of the data and should therefore be unbiased [38],[39]. This resulted in slightly more conservative confidence intervals compared to analytical estimates. This re-sampling strategy was further employed to test for differences in means and variances of the traits between selected sample pairs (*i.e.* Col-wt epiRIL vs. Col-wt, Col-wt vs. Col-*ddm1*, Col-wt epiRIL vs. Col-*ddm1*, etc), yielding a bootstrapped *t* statistic (*t_B_*) and *F* statistic (*F_B_*) and their corresponding *p*-values (*p_B_*), see Tables S2, S3, S4. To test for mean differences we considered the null hypothesis 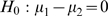 against its alternative 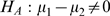. Differences in variances were assessed by testing the null hypothesis 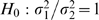 against the alternative 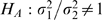, where the subscripts distinguish the two different samples in the comparison.

To decompose the different sources of phenotypic variation in the Col-wt epiRILs, a linear mixed model was fitted. This model took the following form: 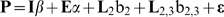, where **P** is the vector of Col-wt epiRIL phenotypic values, **I** represents the design matrix for the fixed-effects intercepts *β* for each of the two greenhouses, **E** is a vector of micro-environmental values (Text S1) with fixed effect α, **L_2_** is the design matrix for the random Line-effect vector b_2_, **L_2_**,**_3_** is the design matrix for the random nested Subline-effect vector b_2_,_3_, and **ε** is the residual error matrix. From the resulting estimates, the variance associated with the Line-effect should be directly interpreted as the portion of total phenotypic variance that is due to epigenetic differences between the lines [40], whereas the Subline-effect estimates the variance due to new DNA sequence mutations or epimutations that may have accumulated independently in the different sublines, gene×environment interactions and maternal effects. All data points exceeding three standard deviations were excluded from the analyses. The *p*-values associated with each of these effects were obtained from hypothesis testing using the likelihood ratio test 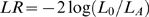, where 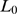 is the likelihood of the full model and 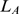 is the likelihood of the reduced model (the full model without the variable of interest). The 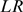 is distributed as a chi-square random variable with the number of degrees of freedom equal to the difference in the number of parameters between the full and the reduced model. The 95% confidence intervals surrounding the parameter estimates were computed from 5000 parametric bootstrap samples. All analyses were performed in R [41].

### Analysis of DNA Methylation, Transcription, and TE Mobilization

DNA and RNA were extracted from seedlings and young rosette leaves, respectively, using DNeasy and RNeasy Qiagen kits, respectively. McrBC (New England Biolabs) digestion was performed on 200 ng of genomic DNA. Quantitative PCR was performed using an ABI 7900 machine and Eurogentec SYBR green I MasterMix Plus on equal amounts of digested and undigested DNA samples. Results were expressed as percentage of loss of molecules after McrBC digestion. Reverse transcription was performed on 1 ug of total RNA using oligodT and Superscript II (Invitrogen). Quantitative PCR was performed as above. Results were expressed as percentage of expression relative to the mean value obtained for three genes (At2g36060; At4g29130; At5g13440) that show invariant expression over hundreds of publicly available microarray experiments. Southern blot analysis of TE mobilization was performed as previously described, using 1 µg of genomic DNA [19],[20].

## Supporting Information

Figure S1 DNA methylation levels measured by McrBC-QPCR. Methylation levels were measured for 14 sequences chosen across the genome. (A) Col-wt and Col-*ddm1*. (B) Example of segregation of differential DNA methylation among the 22 Col-wt epiRILs tested at the F9 generation (BC1-S7). C) Example of loci with non-segregating, wt level DNA methylation among these 22 Col-wt epiRILs.
(0.15 MB PDF)

Table S1 Raw phenotypic data.
(0.37 MB XLS)

Table S2 Estimated population means and variances.
(0.01 MB PDF)

Table S3 Means comparison.
(0.01 MB PDF)

Table S4 Variance comparison.
(0.01 MB PDF)

Table S5 Linear mixed model results.
(0.01 MB PDF)

Text S1 Supporting materials and methods.
(0.26 MB PDF)
