# Supplementary figures and images for: Assessing the Impact of Transgenerational Epigenetic Variation on Complex Traits
Source: PLoS Genet. 2009 Jun 26;5(6):e1000530. doi: 10.1371/journal.pgen.1000530 (PMC2696037; doi:10.1371/journal.pgen.1000530)

A

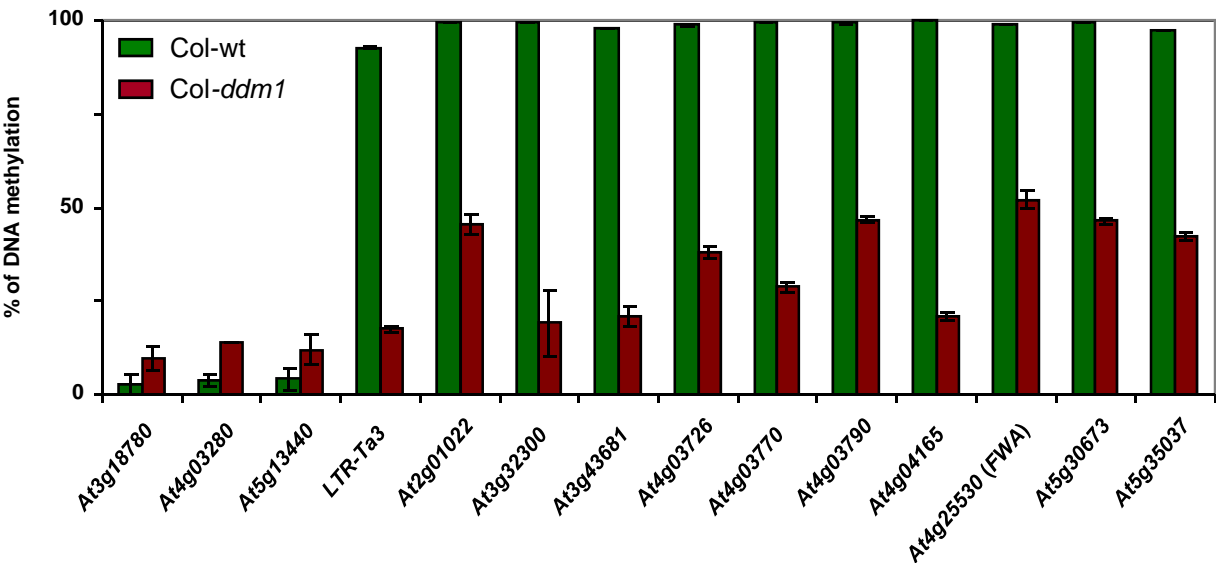

B

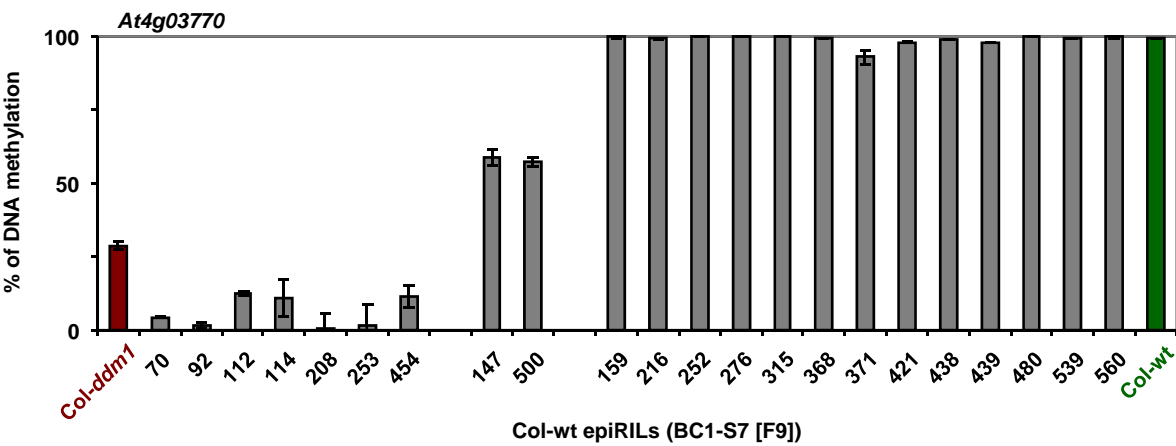

C

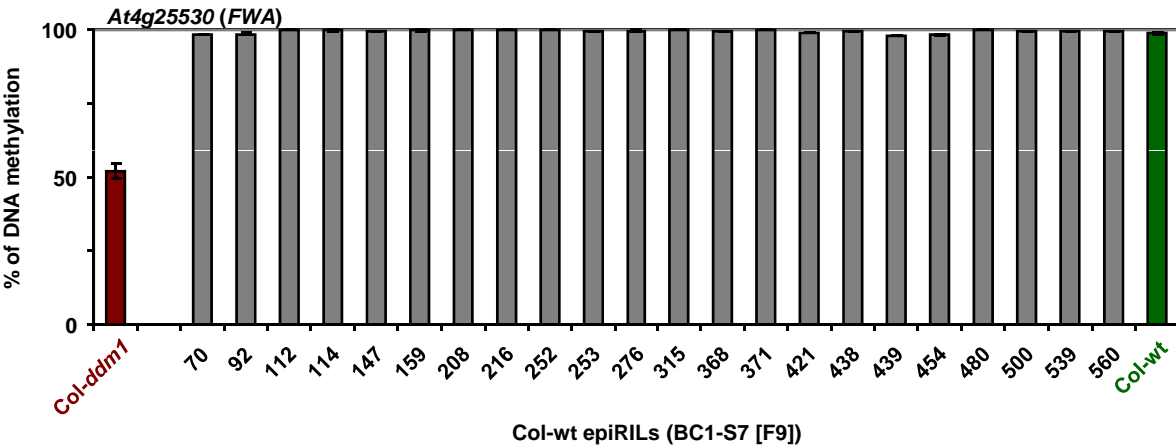

Supplement: Figure S1 — DNA methylation levels measured by McrBC-QPCR. Methylation levels were measured for 14 sequences chosen across the genome. (A) Col-wt and Col-ddm1. (B) Example of segregation of differential DNA methylation among the 22 Col-wt epiRILs tested at the F9 generation (BC1-S7). C) Example of loci with non-segregating, wt level DNA methylation among these 22 Col-wt epiRILs. (0.15 MB PDF) [file pgen.1000530.s001.pdf]
