# Supplementary material for: Assessing the Impact of Transgenerational Epigenetic Variation on Complex Traits
Source: PLoS Genet. 2009 Jun 26;5(6):e1000530. doi: 10.1371/journal.pgen.1000530 (PMC2696037; doi:10.1371/journal.pgen.1000530)
Supplement: Table S2 — Estimated population means and variances. (0.01 MB PDF) [file pgen.1000530.s003.pdf]

Table S2: Estimated population means and variances

Phenotype	Sample	Sample Size	Mean	95 % Cofindence Interval		Variance	95 % Cofindence Interval	
				lower	upper		lower	upper
Flowering time (days)	Col-*ddm1*	200	35.30	34.93	35.70	8.39	6.41	10.56
	Col-wt	199	36.34	35.97	36.72	6.93	5.96	7.83
	Col-wt control	144	39.50	38.92	40.06	8.39	6.45	10.28
	Col-wt epiRIL	3013	37.99	37.79	38.21	11.18	9.37	13.11
Plant height (cm)	Col-*ddm1*	196	38.56	37.79	39.28	30.62	23.66	37.50
	Col-wt	196	48.40	47.87	48.95	15.78	11.56	20.26
	Col-wt control	140	48.40	47.21	49.47	22.49	11.84	35.75
	Col-wt epiRIL	2969	47.84	47.43	48.20	35.53	29.66	43.16

Table S2 provides the numerical results for the estimated population means and variances for flowering time and plants height. The 95% confidence intervals were obtained as described in the Text S1. The results for the Col-wt control population are also included for comparison.
