# Supplementary material for: Assessing the Impact of Transgenerational Epigenetic Variation on Complex Traits
Source: PLoS Genet. 2009 Jun 26;5(6):e1000530. doi: 10.1371/journal.pgen.1000530 (PMC2696037; doi:10.1371/journal.pgen.1000530)
Supplement: Table S3 — Means comparison. (0.01 MB PDF) [file pgen.1000530.s004.pdf]

Table S3: Means comparison

Phenotype	Sample 1	Sample 2	Larger obs. mean	*t*	95 % Confidence Interval		*p* -value
					lower	upper	
Flowering time (days)	Col-wt epiRIL	Col-*ddm1*	Col-wt epiRIL	12.73	10.27	15.26	< 0.0001
	Col-wt epiRIL	Col-wt	Col-wt epiRIL	8.49	6.32	10.85	< 0.0001
	Col-wt epiRIL	Col-wt control	Col-wt control	6.36	3.83	9.29	< 0.0001
	Col-*ddm1*	Col-wt	Col-wt	3.81	2.07	5.26	0.0002
	Col-*ddm1*	Col-wt control	Col-wt control	13.54	11.43	16.04	< 0.0001
	Col-wt	Col-wt control	Col-wt control	10.42	8.13	12.98	< 0.0001
Plant height (cm)	Col-wt epiRIL	Col-*ddm1*	Col-wt epiRIL	22.59	19.81	25.57	< 0.0001
	Col-wt epiRIL	Col-wt	Col-wt	1.83	0.07	3.62	0.07
	Col-wt epiRIL	Col-wt control	Col-wt control	1.92	0.19	5.25	0.18
	Col-*ddm1*	Col-wt	Col-wt	20.36	17.26	23.03	< 0.0001
	Col-*ddm1*	Col-wt control	Col-wt control	17.67	13.77	21.67	< 0.0001
	Col-wt	Col-wt control	Col-wt	0.95	0.03	3.01	0.99

Table S3 provides the comparisons of the phenotypic means between selected sample pairs. The bootstrapped *t*-statistic, 95 % confidence intervals, and the corresponding *p*-values were obtained as described in the text. In each case, the sample with larger observed mean is indicated in column four.
