# Supplementary material for: Assessing the Impact of Transgenerational Epigenetic Variation on Complex Traits
Source: PLoS Genet. 2009 Jun 26;5(6):e1000530. doi: 10.1371/journal.pgen.1000530 (PMC2696037; doi:10.1371/journal.pgen.1000530)
Supplement: Table S4 — Variance comparison. (0.01 MB PDF) [file pgen.1000530.s005.pdf]

Table S4: Variance comparison

Phenotype	Sample 1	Sample 2	Larger obs. var	*F*	95 % Confidence Interval		*p* -value
					lower	upper	
Flowering time (days)	Col-wt epiRIL	Col-*ddm1*	Col-wt epiRIL	1.33	1.01	1.83	0.03
	Col-wt epiRIL	Col-wt	Col-wt epiRIL	1.61	1.30	2.03	0.0002
	Col-wt epiRIL	Col-wt control	Col-wt epiRIL	1.33	1.03	1.84	0.06
	Col-*ddm1*	Col-wt	Col-*ddm1*	1.21	0.91	1.64	0.09
	Col-*ddm1*	Col-wt control	Col-wt control	1.00	0.70	1.36	0.51
	Col-wt	Col-wt control	Col-wt control	1.21	0.90	1.56	0.07
Plant height (cm)	Col-wt epiRIL	Col-*ddm1*	Col-wt epiRIL	1.16	0.86	1.59	0.22
	Col-wt epiRIL	Col-wt	Col-wt epiRIL	2.25	1.62	3.32	0.0001
	Col-wt epiRIL	Col-wt control	Col-wt epiRIL	1.58	0.91	2.93	0.027
	Col-*ddm1*	Col-wt	Col-*ddm1*	1.94	1.34	2.90	< 0.0001
	Col-*ddm1*	Col-wt control	Col-*ddm1*	1.36	0.81	2.73	0.02
	Col-wt	Col-wt control	Col-wt control	1.42	0.70	2.48	0.09

Table S4 provides the comparisons of the phenotypic variances between selected sample pairs. The bootstrapped *F*-statistic, 95 % confidence intervals, and the corresponding *p*-values were obtained as described in the text. In each case, the sample with larger observed variance is indicated in column four.
