# Supplementary material for: Assessing the Impact of Transgenerational Epigenetic Variation on Complex Traits
Source: PLoS Genet. 2009 Jun 26;5(6):e1000530. doi: 10.1371/journal.pgen.1000530 (PMC2696037; doi:10.1371/journal.pgen.1000530)
Supplement: Table S5 — Linear mixed model results. (0.01 MB PDF) [file pgen.1000530.s006.pdf]

Table S5: Linear mixed model results

Phenotype	Model (*N*)	Variable	% variance	95% Confidence Interval		Chi-square	*P*-value
				lower	upper		
Flowering time	2856	G	39.61	39.56	40.01	2289.2	< 0.0001
		M	4.12	3.29	5.05	325.91	< 0.0001
		L	26.41	22.92	31.25	1417.8	< 0.0001
		S	6.49	4.74	8.78	47.104	< 0.0001
Plant height	2813	G	2.45	2.45	2.95	104.92	< 0.0001
		M	0.086	0.0000013	0.379	3.01	0.051
		L	31.88	26.72	37.86	470.99	< 0.0001
		S	3.49	0.54	7.09	2.97	0.053

Table S5 shows the complete numeric results from the linear mixed model analysis. Model (*N*) = Effective sample size; G = Greenhouse, M = Micro-environment variable (see Supplementary Methods); L = Line; S = Subline; % variance = Percent of the variance accounted for by the variable; Chi-square values and the corresponding *p*-values were obtained from hypothesis tests using the likelihood ratio statistic. For each phenotype, outliers > ± 3 standard deviations from the mean were removed from the analyses.
