# Supplementary material for: Assessing the Impact of Transgenerational Epigenetic Variation on Complex Traits
Source: PLoS Genet. 2009 Jun 26;5(6):e1000530. doi: 10.1371/journal.pgen.1000530 (PMC2696037; doi:10.1371/journal.pgen.1000530)
Supplement: Text S1 — Supporting materials and methods. (0.26 MB PDF) [file pgen.1000530.s007.pdf]

Assessing the impact of transgenerational epigenetic variation on complex traits

Frank Johannes, Emmanuelle Porcher, Felipe K. Teixeira, Vera Saliba-Colombani, Matthieu Simon, Nicolas Agier, Agnès Bulski, Juliette Albuisson, Fabiana Heredia, Pascal Audigier, David Bouchez, Christine Dillmann, Philippe Guerche, Frédéric Hospital and Vincent Colot

Text S1. Supporting Materials and Methods

Expected epigenotype frequencies in the presence of contamination

The observed 8% contamination of the *DDM1/DDM1* backcross progeny has the effect that the epigenotype frequencies must be viewed as weighted averages with the backcross contributing a proportion 0.92 and the selfed F1 a proportion 0.08 to the epigenotype frequencies of the subsequent generations. Letting $\pi_{1,\,t=0}$, $\pi_{2,\,t=0}$, and $\pi_{3,\,t=0}$ denote the epigenotype frequencies for, say MM, Mm and mm (M: methylated; m: hypomethylated), in the starting population (t = 0), and introducing the subscript t = 0,1, …, R to denote the subsequent generations in the selfing process, the expected epigenotype frequencies can be expressed for any generation t as

$$\pi_{1,t} = \pi_{1,t=0} + \left(\frac{\pi_{2,t=0} - \pi_{2,t=0}\left(0.5^{t}\right)}{2} \right)$$

$$\pi_{2,t} = \pi_{2,t=0}\left(0.5^{t}\right)$$

$$\pi_{3,t} = \pi_{3,t=0} + \left(\frac{\pi_{2,t=0} - \pi_{2,t=0}\left(0.5^{t}\right)}{2} \right)$$

The table below provides the expected epigenotype frequencies at each selfing generation of the Col-wt epiRIL construction in the presence or absence of contamination at the BC1. It is noteworthy that that the epigenotype frequencies at generation BC1-S6 [F7] are very close in both situations.

8% contamination						No contamination				
t	Generation	freq(MM)	freq(Mm)	freq(mm)		*t*	Generation	freq(MM)	freq(Mm)	freq(mm)
0	BC1[F2]	0.4800	0.5000	0.0200		0	BC1[F2]	0.5000	0.5000	0.0000
1	BC1-S1[F3]	0.6050	0.2500	0.1450		1	BC1-S1[F3]	0.6250	0.2500	0.1250
2	BC1-S2[F4]	0.6675	0.1250	0.2075		2	BC1-S2[F4]	0.6875	0.1250	0.1875
3	BC1-S3[F5]	0.6988	0.0625	0.2388		3	BC1-S3[F5]	0.7188	0.0625	0.2188
4	BC1-S4[F6]	0.7144	0.0313	0.2544		4	BC1-S4[F6]	0.7344	0.0313	0.2344
5	BC1-S5[F7]	0.7222	0.0156	0.2622		5	BC1-S5[F7]	0.7422	0.0156	0.2422
6	BC1-S6[F8]	0.7261	0.0078	0.2661		6	BC1-S6[F8]	0.7461	0.0078	0.2461
7	BC1-S7[F9]	0.72805	0.0039	0.26805		7	BC1-S7[F9]	0.74805	0.0039	0.24805

Experimental conditions and phenotype measurements

The Col-wt epiRILs (N=3030), the Col-wt control lines (N=72), the Col-wt (N=200) and Col-*ddm1* (N=200) parental populations were grown simultaneously in two replicate climate-controlled greenhouses under long day conditions (day: 16h - 20°C/22°C, night: 8h - 16°C/18°C) with complement of artificial light (105 µE/m2 /s) when necessary. For the Col-wt epiRILs, one of the two *BC1-S6* plants for each subline was grown in each greenhouse (ie, 3×505 Col-wt epiRIL plants in each greenhouse). Within each greenhouse, the Col-wt epiRIL plants were randomized over 28 tables ($3x1m^2$). In addition, two or three plants from one of each parental line were systematically placed on each table. Finally, the positions of Col-wt epiRILs and parental lines were randomized within tables. Plants were grown in individual pots ($7x7x7cm^3$) filled with a 90:10 mix of peat and volcanic sand, and topped with a thin layer of granulated cork. About 15 seeds were sown per pot and seedlings were thinned out to retain a single plant that appeared representative of the whole family. Plants were supplemented twice with a nutritive solution during the reproductive phase. Of the planned design, >99 % of plants were available for trait measurements. Flowering time (i.e. number of days between sowing and opening of the first flower) was recorded during plant growth. When plants ceased flowering, they were harvested and stored in herbaria. Plant height was then measured on the dried plants.

Construction of a proxy measure of the micro-environment

The smallest environmental units that were measured in our design are the different tables upon which the Col-wt epiRILs were randomly placed. However, there is reason to believe that many types of environmental influences within a greenhouse can operate on an even smaller scale (e.g. on only one side of the table that is closer to the outside wall of the greenhouse or close to a ventilation fan). In an effort to construct proxy measures of such micro-environmental influences, we took the following approach: We transformed each greenhouse into a Cartesian coordinate system based on its physical dimensions, and noted the metric coordinates of each plant. We then took the standardized phenotypic values of the Col-*ddm1* and Col-wt parental lines (which were represented on each table) as an index of the micro-environmental effect at that position, and then applied a linear interpolation algorithm for irregularly-spaced bivariate data [1,2] to impute standardized values at the positions of each Col-wt epiRIL plant. These imputed values were then interpreted to represent plausible environmental values at those positions, and were subsequently used as a fixed micro-environmental variable for further analyses. Preliminary work showed that this proxy variable explained slightly more of the phenotypic variance than the variable ‘table’ (data not shown). A visual representation of the distribution of micro-environmental influences on the trait flowering time within each of the greenhouses can be seen below.

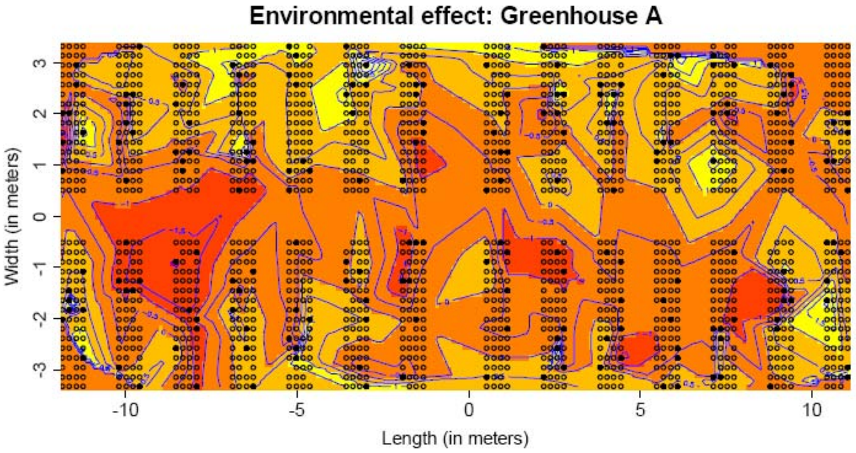

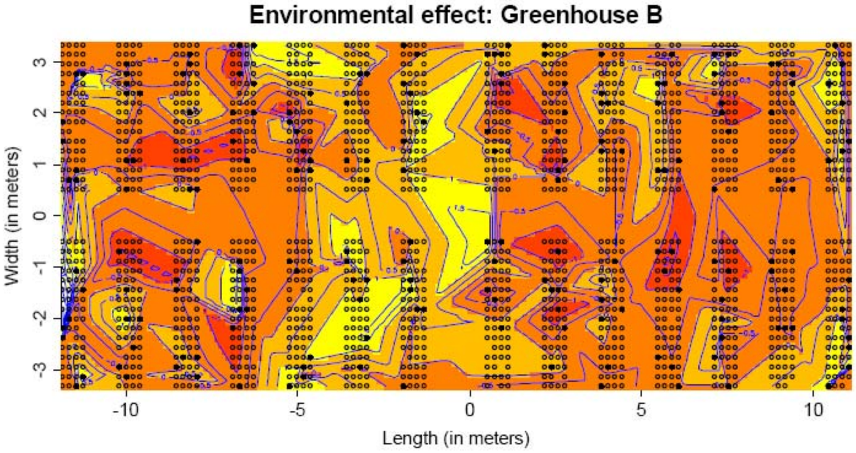

The two figures show the micro-environmental influences on flowering time based on the interpolated parental values. The black circles indicate the positions of either Col-*ddm1* or Col-wt parents which were used for the bivariate interpolation; the open circles denote the positions of the Col-wt epiRILs; red color = low standardized phenotypic values; yellow color = high standardized phenotypic values.

Heritability calculations in the Col-wt control lines

We carried out a separate heritability analysis in the Col-wt control lines (N = 144, 24 lines with 3 sublines each and 2 individuals per subline). The portion of phenotypic variance that is attributable to the variability between the independently selfed lines (H^2, Line-effect) was estimated based on a linear mixed model containing only 'greenhouse' as a fixed intercept and 'Line' as a random effect. The H^2 values thus obtained for flowering time and plant height were 0.0077 and $4.9 \cdot 10^{-8} \approx 0$, respectively. Because the sample size of these control lines was modest, the confidence intervals were quite large, and it was of interested to test whether the broad-sense heritability values (H^2) estimated for these control lines is significantly lower than the H^2 in the Col-wt epiRIL population. To that end, we wished to calculate the statistic $D = H^2_{(epiRILs)} - H^2_{(controls)}$ under the null hypothesis of no differences. To achieve this we used a

stratified bootstrap method. This involved the following steps: 1) Generate random samples of sizes N = 3030 (the sample size of the Col-wt epiRILs) and N = 144 (the sample size of the Col-wt control lines) by sampling first lines and then individuals within lines with replacement and irrespective of sample membership; 2) Estimate heritability $H^2_{B(epiRILs)}$ and $H^2_{B(controls)}$ based on the bootstrap sample and calculate $D_B = H^2_{B(epiRILs)} - H^2_{B(controls)}$; 3) Repeat step 1 and step 2 M times to obtain the boostrap null distribution $g\left(D_B\right)$. The probability of the observed statistic D_{obs} under the null hypothesis of no difference can then be determined empirically as $p_B = \left(\# D_B > D_{obs}\right)/M$, where p_B is the boostrap p-value. The below figure shows $g\left(D_B\right)$ with M = 1000 for flowering time and plant height. The red lines mark off D_{obs}; that is, the observed differences in the broad-sense heritability estimates between the Col-wt epiRILs and the Col-wt control lines.

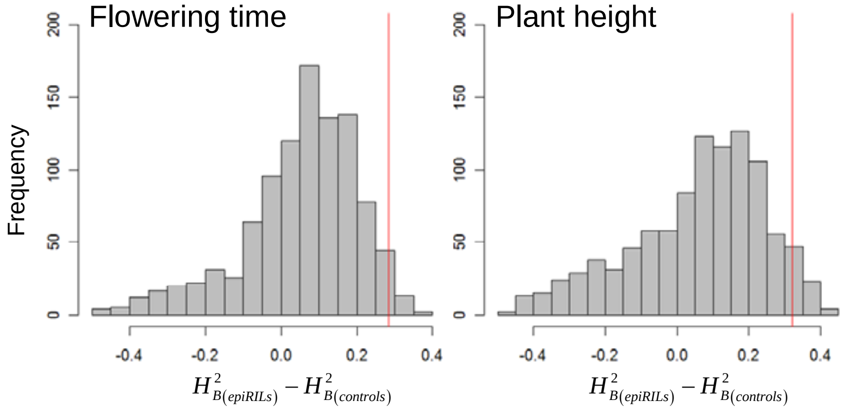

Genotyping of* ddm1-2 *mutation using a dCAPS marker

The region of the *DDM1* locus encompassing the *ddm1-2* mutation [3] was amplified by PCR (35 cycles : 94°C - 30 sec, 58°C - 30 sec, 72°C -1 min) from genomic DNA using primers dCAPS_Col-ddm1_F (5'- ACG AAG CAA CCA AGG AAG AA - 3') and dCAPS_Col-ddm1_R (5'- GAG CCA TGG GTT TGT GAA ACG TA - 3') at 25µM each, in 25 ul reaction volume. Digestion with RsaI (10 U, Invitrogen) was then performed for 2 hours at 37°C directly in the PCR reaction tube, and digests were analyzed on a 2.5% TAE- agarose gel. The dCAPS_Col-ddm1_R primer introduces a mismatch that creates an RsaI site (GTAC) only in the wild type sequence (see figure below).

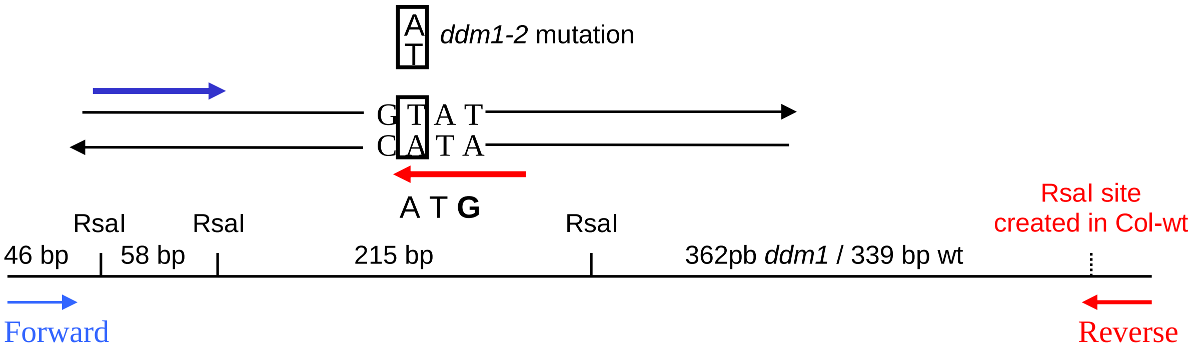

References

1. Akima H (1996) Algorithm 761: scattered-data surface fitting that has the accuracy of a cubic polynomial. ACM Transactions on Mathematical Software 22.
2. Akima H (1978) A Method of Bivariate Interpolation and Smooth Surface Fitting for Irregularly Distributed Data Points. ACM Transactions on Mathematical Software 4: 148-164.
3. Jeddeloh JA, Stokes TL, Richards EJ (1999) Maintenance of genomic methylation requires a SWI2/SNF2-like protein. Nat Genet 22: 94-97.

List of PCR primers

	Chr.	Position	Primer sequences
LTR-Ta3	1	14136849-14137047	5’ TTTGCTCTCAAACTCTCAATTGAAGTTT 3’ ;<br>5’ TAGGGTTCTTAGTTGATCTTGTATTGAGCTC 3’
At2g01022	2	12303-12487	5’ CGAATGAATCCCTTACCCAAC 3’ ;<br>5’ AGCGACATTCGGGAGGAT 3’
At2g36060	2	15149917-15150067	5’ TGAAGTCGTGAGACAGCGTTG 3’ ;<br>5’ GGGCTTCTCCATTGTTGGTC 3’
At3g18780	3	6475949-6476127	5’ GCCATCCCAAGCTGTTCTCTC 3’;<br>5’ CCCTCGTAGATTGGCACAGT 3’
At3g32300	3	13252814-13252997	5’ ACGCCTCCATGTTGTTCCTA 3’ ;<br>5’ TTCTGGAGTCGCGGAAGTAT 3’
At4g03280	4	1440322-1440508	5’ CTCATCCCTTTCCCCTGCTAC 3’ ;<br>5’ TGATTCATCTTCGTTGGCTTC 3’
At4g03726	4	1651320-1651487	5’ GCGGTGAACGTGTCAAATAC 3’ ;<br>5’ GGCAATGGCTTACTGCTAGG 3’
At4g03770	4	1678837-1679004	5’ GGCGCTTATCTCCTGTTCTG 3’ ;<br>5’ ATTTTGGGAAATCGGGAAAC 3’
At4g03790	4	1688923-1689056	5’ TCCCTCGCTGGAGGTATATG 3’ ;<br>5’ AGTCCGCCAACTGATGATGT 3’
At4g04165	4	2009141-2009407	5’ CTGAGGCTCATGAGGTCGAT 3’ ;<br>5’ GGGACACCGTTTCAGCATAC 3’
At4g25530 (FWA)	4	13038394-13038584	5’ GCCATTGGTCCAAGTGCTAT 3’ ;<br>5’ CGGTGCTCGTATGAATGTTG 3’
At4g29130	4	14352280-14352408	5’ GGCGTTTTCTGATAGCGAAAA 3’ ;<br>5’ ATGGATCAGGCATTGGAGCT 3’
At5g13440	5	4308303-4308423	5’ ACAAGCCAATTTTTGCTGAGC 3’ ;<br>5’ ACAACAGTCCGAGTGTCATGGT 3’
At5g30673	5	11414427-11414621	5’ CAGATATTTCCGCACCCG 3’ ;<br>5’ CGCAACAGAGACCCTCAAGT 3’
At5g35037	5	13338092-13338196	5’ TGCTAGATCGAGTGAGTGTCGT 3’ ;<br>5’ CCGAGCCTAGAGAGCAGAAG 3’
